# Jerky Motion of the Reaction Front during Discontinuous Dissolution in a Fe-13.5 at.% Zn Alloy

**DOI:** 10.3390/ma15103525

**Published:** 2022-05-13

**Authors:** Mateusz Chronowski, Jarosław Opara, Boris Straumal, Brigitte Baretzky, Pawel Zięba

**Affiliations:** 1 Institute of Metallurgy and Materials Science, Polish Academy of Sciences, Reymonta St. 25, 30-059 Cracow, Poland; m.chronowski@imim.pl (M.C.); p.zieba@imim.pl (P.Z.); 2 Łukasiewicz Research Network-Institute for Ferrous Metallurgy, K. Miarki St. 12-14, 44-100 Gliwice, Poland; jaroslaw.opara@imz.pl; 3 Karlsruhe Institute of Technology (KIT), Institute of Nanotechnology, Hermann-von-Helmholtz-Platz 1, 76344 Eggenstein-Leopoldshafen, Germany; brigitte.baretzky@kit.edu

**Keywords:** jerky motion, grain boundary, discontinuous dissolution

## Abstract

This paper studies the go- and -stop movement of a receding reaction front (RF) during a discontinuous dissolution (DD) process. A special simulation procedure was applied for the DD reaction to predict a jerky motion of the RF. The Fe-13.5 at.% Zn alloy was selected in which go- and -stop behaviour was revealed in the form of characteristic lines (called “ghost lines”) showing successive positions of receding RF. The results presented for the DD process are quite different from those relevant for the DP reaction at the same Fe-13.5 at.% Zn alloy in terms of go- and -stop motion and movement distance. For the presented case, the go- and -stop periods are relatively long and obtain an order of several dozen seconds, while for the DP reaction, it was only a few seconds. A similar conclusion was formulated after a comparison of the movement distance which, for the DD reaction, is usually longer by 1–2 orders of magnitude. The simulation results of the DD reaction indicate a good agreement with the experimental data presented in the literature for the same dissolution rate. It is necessary to emphasize that the simulation is the only source of data for *z* parameter changes during the -stop period of the DD reaction.

## 1. Introduction

It is well-known that many processes in which the migration of grain or interphase boundaries is involved do not occur in a continuous, steady way. On the contrary, the jerky or equivalently go- and -stop motion is observed. This phenomenon is also confirmed by modelling and simulation procedures. For example, a phase field simulation of the recrystallization process revealed that, if the wavelength of the variations in a deformation microstructure along the grain boundary (GB) is larger than the wavelength of the variations in the direction of migration, parts of the boundary show a go- and -stop type of migration [1]. The same conclusion came from the molecular dynamics and kinetic Monte Carlo simulation of shear motion of GBs [2]. Here, the go- and -stop motion of the tilt GBs has been described in terms of the ratio between the normal motion of the GB and the rigid translation of the adjacent grains.

However, the most spectacular examples of go- and -stop motion come from the examination of discontinuous solid-state phase transformations such as diffusion-induced grain boundary migration (DIGM) [3,4,5], discontinuous precipitation (DP) [6] and discontinuous dissolution (DD) [7,8,9]. The results of such movement appear in the form of so-called “ghost images”, reflecting successive positions of migrating reaction front (in the case of DIGM and DD) after appropriate preparation of metallographic samples or by direct *in situ* observation of the moving RF in the transmission electron microscope (in the case of DP). There is no information in the literature that phase field modelling, molecular dynamics or Monte Carlo simulation were applied to treat this problem for DIGM, DD and DP reactions. The only successful approach has recently been reported for the DP reaction in Al-22 at.% Zn [10] and Fe-13.5 at.% Zn [11] alloys but was based on consideration of mass balance on the moving reaction using the solution of diffusion equation provided by Cahn [12]. The present research was undertaken in order to show whether the same approach is applicable for the DD reaction. As an example, the Fe-13.5 at.% Zn alloy was selected in which go- and -stop behaviour was convincingly revealed in the form of characteristic “ghost lines” showing successive positions of receding reaction front (RF) after careful etching—see Figure 4 in Ref. [13]. One should also note that understanding the kinetics of DP and subsequent DD processes also has a practical aspect as, especially during DP, new high-angle grain boundaries form, which in turn, lead to significant grain refinement without the application of plastic deformation.

## 2. Simulation Procedure

One should emphasize that the simulation of the go- and -stop movement of the RF during the DD process is very similar to the procedure that was applied for the DP reaction [10]. The RF is divided into a fixed number of sectors (~80) along the length of the α phase lamella. Each of the sectors contains a certain number of solute atoms, which reflect the initial state after DP reaction and resulting solute concentration profiles. This means that, contrary to the DP reaction, the sectors are not “empty” at the beginning of the DD process. However, the mass transport along the receding RF of discontinuous precipitates during DD occurs in a similar way as for the DP but in the reverse direction, which was described in detail in Ref. [10].

**Figure 1:**
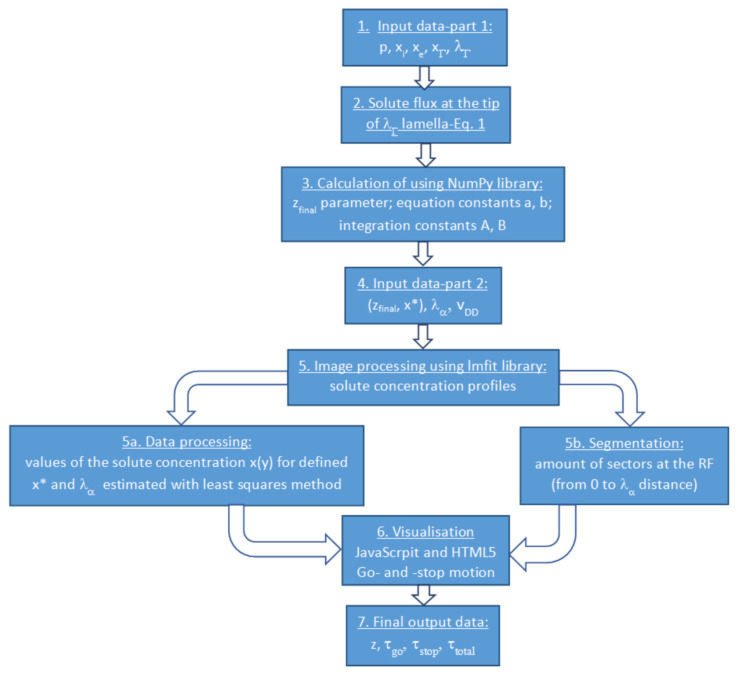
Flowchart of the simulation procedure.

The input data (Step 1 in Figure 1) for the simulation are *p*, *x_o_*, *x_i_*, *λ_α_* and *λ_Γ_*, which describe the properties of the material after DP. Here, *x_o_* is the initial solute concentration in the alloy; *x_i_* is the solute concentration in the α lamella at the α/Γ interface; and *λ_α_* and *λ_Γ_* are the thicknesses of the α lamella and Γ lamella, respectively. The range of values relevant for *p* and *λ_α_* were taken from Ref. [14]—Table 1—for *x_o_* = 13.5 at.% Zn. They were determined using Cahn’s model [12] of DP reaction, which is based on balancing the solute atom masses affected by diffusion along the RF and the motion of the RF. The thickness of the Γ lamella was calculated from the lever rule and adjustment factor 1.14–1.23.

In Step 2 (see Figure 1), the equation resulting from the consideration of the solute flux leaving the tip of Γ lamella during the DD reaction is used in the form provided by Zięba and Pawłowski [15]:

(1) $$\lambda_{\Gamma }x_{\Gamma }\;=\;\frac{2}{z}\left[\left(x*\;-\;x_{o}\;-\;\frac{z^{2}\left(x_{o}\;-\;x_{1}\right)}{p^{2}\;-\;z^{2}}\right)\tanh(z\lambda_{a}/2)\;+\;\frac{pz\left(x_{o}\;-\;x_{1}\right)\tanh(p\lambda_{a}/2)}{p^{2}\;-\;z^{2}}\right]$$

where $p\;=\;\left(\frac{v_{DP}}{s\delta D_{b}}\right)$.

Here, x* is the solute concentration in the newly formed α~ solid solution at the tip of the solute rich Γ lamell, *v_DP_* is the rate of DP, *s* is the segregation factor, *δ* is the grain boundary width, and *D_b_* is the grain boundary diffusion coefficient but relevant for DP. Note, that *p* is related to Cahn’s parameter in his original approach [12] by C = *p*^2^ (*λ_α_*)^2^. Equation (1) allows the *z* parameters describing kinetics of the DD to be calculated by the iteration procedure for the arbitrarily selected values of x* concentrations.

In Step 3 (Figure 1), the calculated (x*, *z*) pairs are inserted into a main program simulating the go- and -stop movement in order to verify whether they result in a realistic solution. In this way, the estimated *z_final_* parameter corresponds to the beginning of the stop period. Having *z_final_* and x* pairs, it is possible to determine the *A*, *B*, *a* and *b* constants in Equation (2) and, consequently, the *x*(*y*) solute concentration profiles formed due to discontinuous dissolution.

(2) $$x(y)\;=\;A\sinh(zy\lambda_{a})\;+\;B\cosh(zy\lambda_{a})\;+\;\frac{a}{p^{2}\;-\;z^{2}}\;\cosh(py\lambda_{a})\;-\;\frac{b}{p^{2}\;-\;z^{2}}\sinh(py\lambda_{a})\;+\;x_{o}$$

where

(3a) $$z\;=\;{\left(\frac{v_{DD}}{s\delta D_{b}}\right)}^{\frac{1}{2}},\;A\;=\;-B\;\cdot \;\tanh(z\lambda_{a}/2)$$

(3b) $$B\;=\;x*\;-\;x_{o}\;-\;\frac{a}{p^{2}\;-\;z^{2}}$$

(4a) $$a\;=\;z^{2}\left(x_{o}\;-\;x_{1}\right)$$

(4b) $$b\;=\;a\tanh(p\lambda_{a}/2)$$

Here, *v_DD_* is the rate of DD and *y* is a normalized co-ordinate measured from the edge of the solute rich Γ lamella in the direction perpendicular to the α lamella. Note that *D_b_* in Equation (2) is the grain boundary diffusion coefficient for the *DD* reaction. The Equation (2) results from the solution of diffusion problem are exactly the same as those for DP [12] but occurring in reverse direction.

Subsequently, Steps 4 and 5 (see Figure 1) of the simulation can start for the various values of *λ_α_* and *v_DD_*. The “Segmentation” box makes it possible to estimate the number of solute atoms that are present in particular sectors along the width of α lamella after a certain time, and the corresponding distance has been covered by the receding RF. If the number of atoms exceeds the limit value and the RF stops, then the “Segmentation” box shows how fast the relaxation process occurs. Simultaneously, the “Data processing” box provides relevant solute concentration profiles and resulting values of *z* parameters. Everything is visualized (Step 6 in Figure 1) in graphical form and, except for *z* parameters, delivers τ*_go_*—the time during which RF engages in backward movement and the corresponding movement distance—as well as τ*_stop_*—the time during which the RF is immobile. The developed program utilizes several programming languages, such as JavaScript, HTML5 and Python. JavaScript and HTML5 are mainly for simple, graphical visualization, while Python with the “lmfit” library was used to calculate and fit the data.

One should emphasize that the simulation procedure shows some limitations. Reasonable results can be obtained for the following maximum values of parameters: *λ_α_* = 300 nm, *v_DD_* = 450 nm/s and C = 7.23. The minimum values are not very critical and correspond well with those published previously [10,11].

## 3. Results

As was already mentioned, the physicochemical state of the material just before the beginning of the DD process is different in comparison to the beginning of the DP reaction. This is not a supersaturated solid solution with the same solute content but the lamellar microstructure of solute-rich and solute-depleted lamellae. Therefore, it is necessary to find out how the DP state described by the *p* (or C), *x_o_*, *x_i_*, *λ_α_* and *λ_Γ_* parameters influences the subsequent DD reaction.

**Figure 2:**
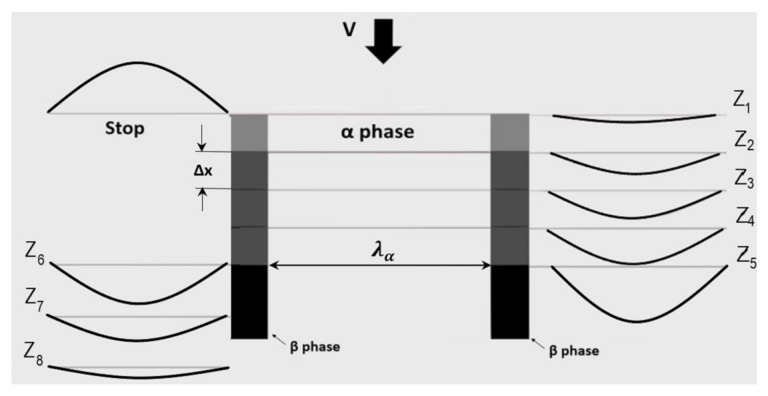
Schematic view of the growth of a single cell during DD with accompanying solute concentration profiles in the go- and -stop period for Cahn’s parameter C = 7.23 and x* = 16 at.% Zn. *v_DD_* = 0.5 nm/s, *λ_α_* = 150 nm.

A visualization of the whole process is shown in Figure 2. The starting solute concentration profile after DP reaction is shown on the left side of the graph with a characteristic reverse “u” shape and corresponding C parameter. On the other hand, the “u” shape profiles and parameters z_1_–z_5_ describe the go- period of DD, while z_6_–z_8_ are relevant for the stop period.

Table 1 summarizes the results of the simulation for two extrema (C_min_ = 1.74 and C_max_ = 7.23) and an arbitrarily selected x* = 16 at.% Zn. The values of α lamella thickness and receding rate were taken as λ_α_ = 150 nm and *v_DD_* = 0.5 nm/s. They clearly indicate that the input parameters of DP show almost negligible influence on the resulting *z* parameters characterizing the DD reaction. Moreover, the extension of the range of x* up to 25 at.% Zn did not show any significant differences in the go- period (τ*_go_*), -stop period (τ*_stop_*) or movement distance (Table 2). The same holds true for other values of *λ_α_* and *v_DD_*. Therefore, the whole simulation was performed for the C = 7.23 at.% Zn.

Table 3 shows the results obtained for *λ_α_* = 150 nm, x* = 16 at.% Zn and various values of *v*. One can see that the parameter *z* increases slightly during the go- period, reaching its maximum at the point at which the receding RF stops. Then, the gradual decrease in *z* parameter is observed up to the values, which correspond well with those at the beginning of the go- period.

Table 4 summarizes all the results from the simulation in terms of the go- (τ*_go_*) and stop- (τ*_stop_*) times; the distances covered by the receding RF; and the maximum values of the *z* parameters, which correspond to the end of the go- periods. One can see that, along with the increase in dissolution rate (*v_DD_*), time of movement (τ*_go_*) and time during which the RF remains immobile (τ*_stop_*) decrease but the movement distance increases. On the other hand, the increase in Zn content in the dissolved area close to the tip of the Γ lamella (x*) lead to an increase in τ*_go_* and the movement distance, while the changes in τ*_stop_* did not show any pronounced tendency.

The increase in α lamella thickness leads to increases in τ*_go_*, τ*_stop_* and movement distance, but this tendency vanishes along with the increase in x* concentration. The larger the α lamella thickness, the larger the values of *z* parameter, regardless of the value of x*.

## 4. Discussion

It is necessary to note that the results presented in Table 4 are quite different from those relevant for the DP reaction at the same Fe-13.5 at.% Zn alloy in terms of τ*_go_*, τ*_stop_* and movement distance. In the present case, τ*_go_* and τ*_stop_* are in the order of several dozen seconds except for the highest receding rate at which τ*_stop_* is less than ten seconds.

For comparison, τ*_go_* and τ*_stop_* for the DP reaction did not exceed 2 s and, in most cases, was even less than 1 s [11]. A similar remark can be made after comparison of the movement distance. Again, for the DP reaction, movement distance was not longer than 40 nm [11], while it was usually longer by 1–2 orders of magnitude for the DD reaction.

One has to emphasize that referring to the experimental data obtained for Fe-13.5 at.% Zn alloy aged at 723 K and subsequently subjected to the DD reaction at 886 K [9], the average distance between two neighbouring “ghost lines” was found to be ca. 0.2–0.3 μm for the *v_DD_* = 5 nm/s, which is in good agreement with our results for the same dissolution rate. Moreover, τ*_go_* = 40–60 s calculated from these data fits well with τ*_go_* reported in Table 4.

Other references can be made for the DD reaction in CuCd alloys examined by Sulonen [8]. Here, the movement distance measured at light microscopy micrographs was ca. 1.0–1.25 μm, which fits well with the data for *v_DD,_* ranging from 0.5 to 50 nm/s as listed in Table 4. Hackney et al. [16] investigated the DD reaction in Pb-3.23 wt.% Sn alloy. Seven “ghost lines” left by the receding RF at the distance of ca. 8.5 μm can be distinguished, which result in one go- event equal to slightly more than 1 μm. Unfortunately, the lack of data related to *v_DD_* does not allow for any conclusions about τ*_go_*. Other research performed by den Broeder [4] for the DIGM process in Cu_90_Zn_10_/Cu couples annealed at 350 °C provided the values of individual go- events close to 0.3–0.4 μm.

It is quite clear that the higher the *v_DD_*, the shorter the time for the diffusion during displacement of RF by its width. Therefore, the faster receding RF is “clogged” with excessive Zn atoms that did not have enough time to be dissolved in the solid solution. Consequently, the time intervals for go- and -stop events are shorter.

The same effect results from the larger thickness of α lamella. Here, the increase in the diffusion distance (λ_α_/2) which has to be covered by the receding reaction front overlaps with the increasing *v_DD,_* although this is not a very strong influence.

Additionally, the increase in go- time along with the increase in x* concentration can be explained if the change in Zn content in the place where the Γ lamella previously existed is lower than the time after which the RF is “clogged” is longer. This results from the simple deduction for 68–25 at.% Zn instead of 68–16 at.% Zn, where 68% is the average content of zinc in Γ lamella. The stop time is not very sensitive to the changes in x*, and it takes more or less the same values for certain set (*v*_DD_, x*) parameters except for the smallest *v_DD_*. The origin of such behaviour is unknown, and it can only be speculated that this may be associated with some features of a relaxation process preceding another go- stage.

## 5. Conclusions

The simulation of the DD reaction in the Fe-13.5 at.% Zn alloy leads to the following conclusions:

1. The Zn concentration profile (existing in the α phase lamellae after the former DP process) has only a little influence on the occurrence of this phase transformation and can be neglected in the simulation procedure.
2. The DD reaction takes place by a jerky motion of the receding reaction front, which can be described as subsequent go- and -stop events.
3. The time of the go- and -stop periods is relatively long and decreases with an increase in dissolution rate. The opposite tendency holds true for the values of movement distances and *z* parameters.
4. The time of a go- event is longer for higher values of x* left behind the receding tip of the Γ lamellae. On the other hand, the stop period is mostly dependent on x* values for the constant dissolution rate.
5. The simulation is the only source of data for *z* parameter changes during the stop period of the DD reaction. Knowledge of the z parameter values makes it possible to restore the Zn concentration profiles left in the post-dissolution area.

## Figures and Tables

**Table 1:** Values of z parameters for one cycle of go- and -stop motion and two extreme *C* parameters.

Stage of Motion	z × 10^6^ (m^−1^)	C Parameter	
		C_min_ = 1.74	C_max_ = 7.23
Go	z_1_	1.03	1.20
	z_2_	1.11	1.32
	z_3_	1.22	1.45
	z_4_	1.51	1.66
	z_5_	1.97	1.80
stop	z_6_	1.59	1.44
	z_7_	1.29	1.29
	z_8_	1.05	111

**Table 2:** Results of the simulation for λ_α_ = 150 nm, *v_DD_* = 0.5 nm/s and various values of x*.

C_max_ = 7.23			
Zn-content at the β tip (at.% Zn)	x* = 16	x* = 20	x* = 25
τ*_go_* (s)	15	20	31
τ*_stop_* (s)	8	12	19
Movement distance (μm)	5.3	6.1	6.9
Parameter z (1/m)	1.8 × 10^6^	1.84 × 10^6^	1.95 × 10^6^
C_min_ = 1.74			
Zn-content at the β tip (at.% Zn)	x* = 16	x* = 20	x* = 25
τ*_go_* (s)	15	22	38
τ*_stop_* (s)	8	14	22
Movement distance (μm)	5.4	6.2	7.3
Parameter z (1/m)	1.97 × 10^6^	2.02 × 10^6^	2.14 × 10^6^

**Table 3:** Results of the simulation for λ_α_ = 150 nm, x* = 16 at.% Zn and various values of *v_DD_*.

Stage	Time (s)	Parameter z × 10^6^ (m^−1^)	Movement Distance (μm)
*v* = 420 nm/s, λ_α_ = 150 nm			
Go	1	1.04	0.42
	2	1.10	0.84
	3	1.18	1.26
	4	1.24	1.68
	5	1.30	2.10
	6	1.49	2.52
	7	1.57	2.94
	8	1.66	3.36
	9	1.79	3.78
	10	1.93	4.20
Stop	11	2.11	4.62
	12	1.89	4.62
	13	1.56	4.62
	14	1.36	4.62
	15	1.22	4.62
	16	1.11	4.62
	17	1.00	4.62
*v* = 50 nm/s, λ_α_ = 150 nm			
Go	5	1.14	0.25
	10	1.28	0.50
	15	1.34	0.75
	20	1.44	1.00
	25	1.59	1.25
	30	1.68	1.50
Stop	35	1.99	1.75
	40	1.76	1.75
	45	1.65	1.75
	50	1.43	1.75
	55	1.23	1.75
	60	1.11	1.75
*v* = 5 nm/s, λ_α_ = 150 nm			
Go	10	1.16	0.05
	20	1.30	0.10
	30	1.50	0.15
Stop	40	1.83	0.20
	50	1.49	0.20
	60	1.26	0.20
	70	1.13	0.20
*v* = 0.5 nm/s, λ_α_ = 150 nm			
Go	10	1.20	0.005
	20	1.32	0.010
	30	1.45	0.015
	40	1.66	0.020
Stop	50	1.80	0.025
	60	1.44	0.025
	70	1.29	0.025
	80	1.11	0.025

**Table 4:** Summary of go- and -stop motion for λ_α_= 150 and 300 nm; *v_DD_* = 0.5–420 nm/s; and x* = 16, 20, and 25 at.% Zn. Fe-13.5 at% Zn alloy, C_max_ = 7.23.

Receding Rate (nm/s)	Thickness of λ Lamella λ_α_ = 150 nm											
	x* = 16 at.% Zn				x* = 20 at.% Zn				x* = 25 at.% Zn			
	Go (s)	Stop (s)	Distance (μm)	z × 10^6^ (m^−1^)	Go (s)	Stop (s)	Distance (μm)	z × 10^6^ (m^−1^)	Go (s)	Stop (s)	Distance (μm)	z × 10^6^ (m^−1^)
**0.5**	50	30	0.025	1.80	60	50	0.03	1.84	80	40	0.04	1.95
**5.0**	40	30	0.20	1.83	60	40	0.3	1.9	70	40	0.35	1.99
**50**	35	25	1.75	1.99	40	25	4.0	2.0	45	25	4.5	2.11
**420**	11	6.0	4.62	2.11	14	5.0	5.88	2.26	16	7.0	6.72	2.34
	**Thickness of λ Lamella λ_α_ = 300 nm**											
**0.5**	53	32	0.0265	1.84	62	51	0.031	1.93	85	40	0.0425	2.13
**5.0**	47	36	0.235	1.91	60	43	0.300	2.00	79	40	0.395	2.24
**50**	38	28	1.9	2.02	43	28	2.15	2.12	49	25	2.45	2.44
**420**	12	7.2	5.04	2.13	18	6.4	7.56	2.35	19	7.0	7.98	2.77

## Data Availability

The data are contained within the article.
